# Comparative Analysis of the Properties of Acid-Base Indicator of Rose (*Rosa setigera*), Allamanda (*Allamanda cathartica*), and Hibiscus (*Hibiscus rosa-sinensis*) Flowers

**DOI:** 10.1155/2015/381721

**Published:** 2015-12-27

**Authors:** Stanley I. R. Okoduwa, Lovina O. Mbora, Matthew E. Adu, Ameh A. Adeyi

**Affiliations:** ^1^Directorate of Research and Development, Nigerian Institute of Leather and Science Technology (NILEST), Zaria 810001, Kaduna State, Nigeria; ^2^Directorate of Science Laboratory Technology, NILEST, Zaria 810001, Kaduna State, Nigeria; ^3^Department of Medical Laboratory Services, Antiretroviral Therapy Centre, Central Hospital, Agbor 321251, Delta State, Nigeria

## Abstract

The need to develop effective alternative for synthetic indicators is the demand of present-day chemistry. The acid-base indicator properties of Rose (*Rosa setigera*), Allamanda (*Allamanda cathartica*), and Hibiscus (*Hibiscus rosa-sinensis*) flowers were examined. Colour pigments were extracted from the flowers via cold and solvent extraction using soxhlet extractor. The pH value of the extracts with wavelengths of absorption was determined using ultraviolet spectrophotometer. From the results obtained, all the extracts exhibited sharp contrast between their colours in acid and base. Their pH was found to be 5.5 for cold extract of Rose and 5.6 for solvent extraction, 5.24 for cold extract of a Hibiscus and 6.52 for solvent extraction, 5.35 for cold extract of Allamanda, and 5.45 for solvent extraction. The maximum wavelengths of absorption obtained for all the extract fall within the visible region of electromagnetic spectrum. These values are almost similar to that obtained from synthetic indicators. It is on these bases that we concluded that natural indicators could be an excellent replacement for synthetic indicators since they are cheap, readily available, simple to extract, not toxic, user and environmentally friendly.

## 1. Introduction

Despite the presently available instrumental techniques for the chemical analyses of various samples, the conventional methods of analyses are still appropriate in most applications. A number of conventional analytical techniques presently in use today include the gravimetry and titrimetry. In titrimetry, the equivalence point is usually determined by the end point in the titration. The end point in traditional titrimetry is more often than not indicated by some substances added into the analyte solution, which change colour right away after the equivalence point has been attained. These substances are known as indicators [1–3].

Indicators are pigments or dyes that can be isolated from a variety of sources, including plants, fungi, and algae [4, 5]. Virtually any flower that is red, blue, or purple in colour contains a class of organic pigments known as anthocyanin that can change colour with pH [6]. Some naturally coloured substances change colours when the acidity or alkalinity of their environment changes, for example, grape juice, brown tea, and some flower pigments. These substances are called acid/base indicators [7]. Indicators change colour at a particular stage of chemical reaction [8]. A number of commonly used indicators in the laboratories are methyl red, methyl orange, phenolphthalein, phenol red, methyl yellow, pentamethoxy red, bromophenol blue, thymol blue, and so forth [9]. Most type indicators are available for different types of titrimetric analyses. For acid-base titrations, organic dyes, which are either weak acids or bases, serve excellently as indicators [1, 3].

Acid-base indicators are substances that are revealed through the characteristic colour which changes the degree of acidity or alkalinity of solutions [10, 11]. The choice of an indicator for a particular titration depends on the characteristic of the neutralization curve. In acid-base titration, an indicator is used to determine the end point of the titration at which the acid and base are in the exact proportions necessary to form salt and water only. Consider

(1) $$\begin{array}{c} {\text{H}}^{+}{\text{Cl}}^{-}+{\text{Na}}^{+}{\text{OH}}^{-}⇄{\text{H}}_{2}{\text{O}}_{\left(\text{l}\right)}+{\text{NaCl}}_{\left(aq\right)} \end{array}$$

Majority of indicators in use today are synthetic. A synthetic indicator is man-made chemical substance in the laboratory which is used to determine pH of a substance, such as litmus paper [12, 13]. Litmus paper contains naturally occurring substances that indicate pH levels, but the item as a whole is made in the laboratory such as methyl red, methyl orange, phenolphthalein, phenol red, methyl yellow, pentamethoxy red, bromophenol blue, and thymol blue. Synthetic indicators have certain disadvantages such as high cost, availability, and chemical pollution; hence natural indicators obtained from various plant parts like flowers, fruits, and leaves will be more advantageous [14, 15]. In addition, some of these synthetic indicators have toxic effects on users such as diarrhea, pulmonary edema, hypoglycemia, and pancreatitis and they can result in abdominal cramps, skin rash, eruptions, erythema, and epidermal necrosis and cause environmental pollution [16, 17].

On the bases of these rationales of the hazardous effects of synthetic indicators, there has been an increasing interest in the search for alternative sources of indicators from natural sources of plant origin. These alternatives from plant origin are probably cheaper, readily available, easy to extract, less toxic to users, and environmentally friendly [18–21]. Quite a number of dyes are obtainable from natural products. For instance, in Nigeria, several researchers have extracted different type of dyes from a variety of local plants [1, 16, 22]. Also, several studies by various investigators have reported the effectiveness of natural indicators in acid-base titrations [16, 22]. Some flowers such as Rose, Allamanda, and Hibiscus work in nature like litmus paper, changing colour in the presence of acids or bases; these flowers are usually mildly acidic or alkaline themselves, and they change colour when mixed with a substance that has an opposite pH [23]. Others are plants and leaves such as red cabbage extract, blueberry juice, black tea, beet juice, rhubarb, and tomato leaves [23].

An indicator does not change colour from pure acidic to pure alkaline at specific hydrogen ion concentration, but rather colour change occurs over a range of hydrogen ion concentrations. This is the colour change interval expressed as the pH range. A natural indicator is a natural substance usually from plant origin that can be used to determine the pH of another substance [6, 21]. Hence in this research, we aim to evaluate the properties of some natural substances such as Rose (*Rosa setigera*), Allamanda (*Allamanda cathartica*), and Hibiscus (*Hibiscus rosa-sinensis*) flowers in order to ascertain their analytical potentials as indicators.

## 2. Methodology

### 2.1. Materials

The materials are Allamanda flowers, Rose flowers, Hibiscus flowers, distilled water, methanol, sodium hydroxide, and concentrated hydrochloric acid.

### 2.2. Apparatus

The apparatus consists of mortar and pestle, weighing balance, beakers, conical flask, burette, pipette, retort stand with clamp, white tile, wash bottle, spatula, stirrer, soxhlet extractor, and filter paper (whatman 40).

### 2.3. Sample Preparation

The three different samples collected were free from unwanted materials (pistil, stamen, and stalk). They were dried at room temperature. The weights of the samples were constantly taken to ensure that the samples are completely dried. The samples were grounded with mortar and pestle. Each of the samples was filtered and 20 grams was weighed for each and extracted with distilled water and methanol.

### 2.4. Sample Extraction

The three flowers, Rose, Hibiscus, and Allamanda, were extracted by two main methods, namely, soxhlet extraction method and cold method of extraction [24].

#### 2.4.1. Soxhlet Extraction

Exactly 20 grams of each sample of Rose, Hibiscus, and Allamanda was weighed into a paper and it was wrapped and placed inside a soxhlet apparatus. A condenser and a round bottom flask were fitted to the extractor; 250 mL of methanol was placed in the extractor and the temperature was set to 65°C, that is, the boiling point for methanol. The colouring matter of the sample was allowed to continue siphoning until the solvent becomes colourless. The sample was then removed from the extract to allow the extracting solvent to be recovered. The extract was poured into an evaporating dish and allowed to dry on the water bath. It was then placed inside the oven for further drying and then kept in desiccators to cool [25].

#### 2.4.2. Cold Extraction

Exactly 20 grams of each sample of Rose, Allamanda, and Hibiscus was weighed and transferred into three separate beakers; 250 mL of distilled water was added into each sample and left overnight. On the following day, they were decanted into clean beakers and rinsed with 20 mL of water in order to clear out the colouring matter. It was then concentrated on a water bath [26].

### 2.5. Characterization of Extract

The extracts were characterized with the use of UV/Visible spectroscopy to determine the wavelength of maximum absorption.

#### 2.5.1. Reaction of the Extracts with Acids and Bases

Samples of the extract obtained were added to different acids and bases to test if there will be any colour change. The acids used for these were H_2_SO_4_ and CH_3_COOH, while the bases used were NaOH and KOH.

#### 2.5.2. Acid-Base Titration with Extract as Indicator

Titrations were carried out using 0.1 M NaOH and 0.1 M H_2_SO_4_ for strong acid-strong base titration. 0.1 M solution of CH_3_COOH and 0.1 M NaOH were used for weak acid strong base titration. The accuracy of the end point for all experimental samples and trials was repeated three (3) times to check the precision and reliability.

#### 2.5.3. Ultraviolet (UV) Spectroscopy

The Jenway 6305 spectrophotometer was used; the cell to be used for the UV/Visible spectroscopy was washed thoroughly with distilled water. Distilled water was used to calibrate the instrument at the wavelength of 400 nm. Therefore 0.001 mL of each extract was diluted with 10 mL of distilled water and 5 mL of the extract was measured and placed in the cell. The absorbance of the extract was determined within the visible region (i.e., 400–750 nm) and the wavelength of maximum absorption (ʎ_max⁡_) of each extract was extrapolated from the graph.

## 3. Result and Discussion

The results obtained for the evaluation of the extracts are as presented in Tables 1, 2, 3, 4, 5, 6, 7, and 8.

From the two methods used in the extraction of the flowers, solvent extraction was so far the best method because more dye was obtained especially for hibiscus which is sticky and slippery in water (Table 1); it becomes very difficult to separate the dye from the residue. In a likewise manner, comparing the two methods of extraction, the extracts/standard indicators reaction with the same acids and bases were similar (Tables 2–8). For any acid-base titration, the indicator chosen should be such that pH of such an indicator is closer to the equivalence point at pH 7.

Furthermore, the titration of weak acid and strong base has an equivalence point lower than pH 7 and the extracts can also fit such a titration. Allamanda methanol extract when titrated against a strong acid had similar titre value as phenolphthalein and methyl red and therefore can serve as alternative for this indicator. This was similar to the reports of Eze and Ogbuefi [27] and other investigators that did related work on indicators [3–6], but there was a slight difference in the result as compared with that of Singh et al. [13]. The Hibiscus extract had similar tire value with phenolphthalein and methyl red and therefore can as well replace these two commercial indicators. The observations derived from the present research on* Rosa setigera*,* Allamanda cathartica*, and* Hibiscus rosa-sinensis* flowers also corroborate that of other investigators with respect to indicators extracted from plants [1, 4–6, 11, 27, 28]. Besides the use of flowers for the extraction of natural indicators, other parts of plant have been explored recently [16, 22, 29, 30].

**Figure 1:**
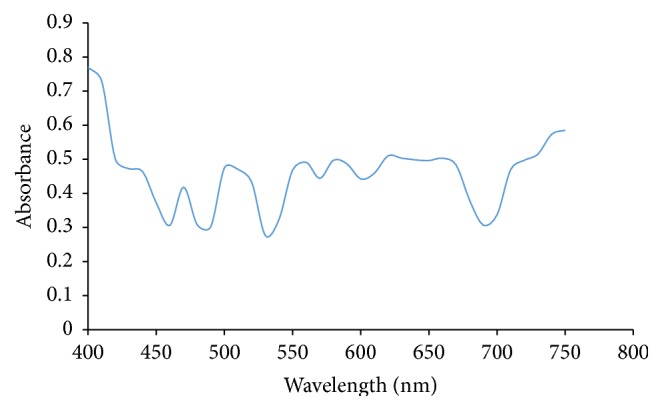
Allamanda distilled water extract.

**Figure 2:**
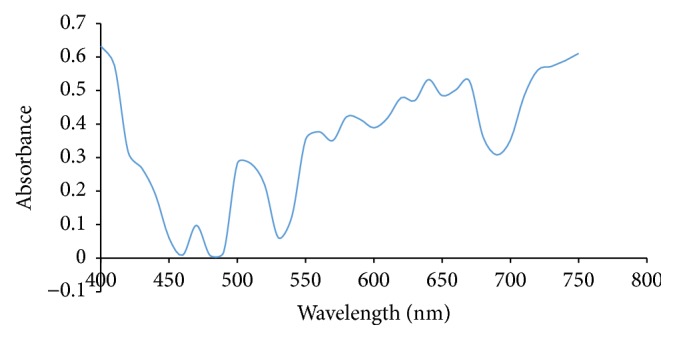
Allamanda methanol extract.

**Figure 3:**
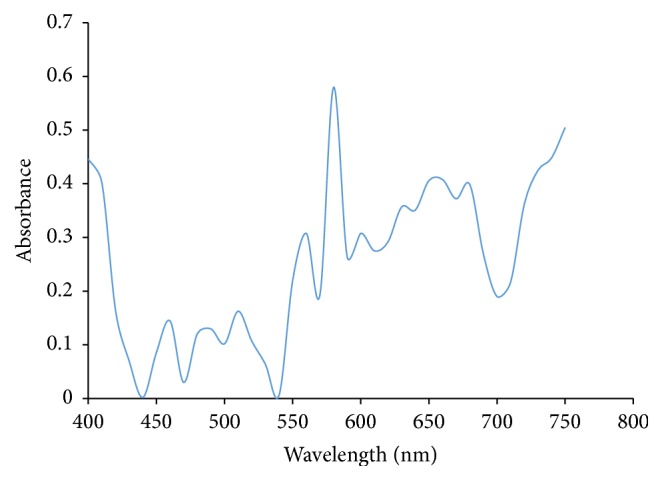
Hibiscus distilled water extract.

**Figure 4:**
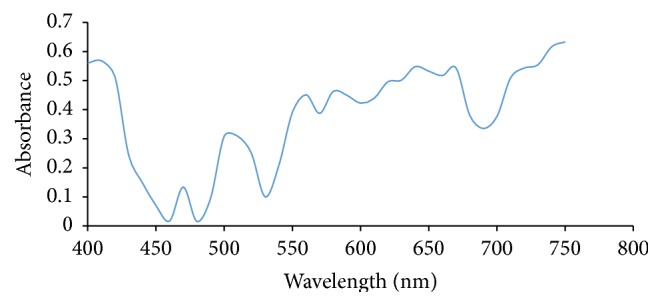
Hibiscus methanol extract.

**Figure 5:**
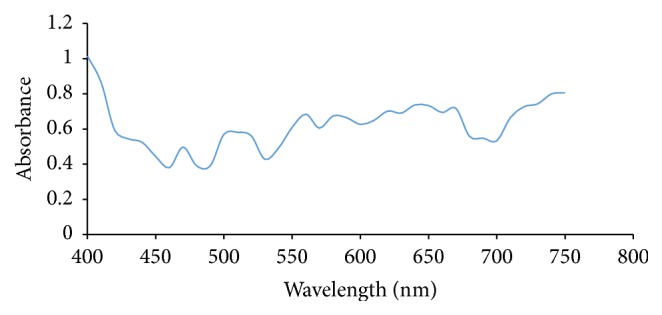
Rose distilled water extract.

**Figure 6:**
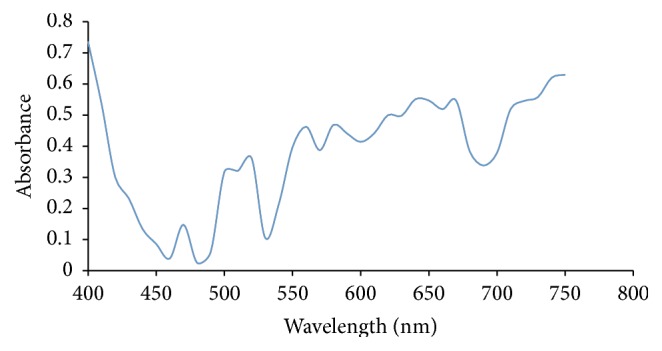
Rose methanol extract.

Ultraviolet/Visible Spectroscopy was carried out on each extract of Rose, Allamanda, and Hibiscus and the absorbance was plotted against wavelength. From Figures 1 and 2, it showed that Allamanda extract using water and methanol absorbs at ʎ_max⁡_ of 620 nm and 640 nm, respectively. Figures 3 and 4 showed that Hibiscus extract for both water and methanol absorbed at ʎ_max⁡_ of 580 nm and 640 nm, while Figures 5 and 6 showed that Rose extract for both water and methanol absorbed at ʎ_max⁡_ of 640 nm and 670 nm, respectively. Therefore the spectra of the extracts show that they have more than one peak indicating the different components present in each of the samples and at visible regions.

## 4. Conclusion

The results obtained from the present study reveal that the analytical potential of the dye extracts is very promising as seen in its application in acid-base titrimetry where it was discovered to perform best in strong acid-strong base titration compared to weak acid strong base with a sharp and clear colour change from yellow to brown for Rose extract, from yellow to colourless for Allamanda extract, and from pale yellow to colourless for Hibiscus extract. The two extracts gave clear colour change with acids and bases and the colour change was maintained with different acids and bases. The sharp contrast between their colours in acid and base made the pigment suitable for use as acid-base indicators. Also for the fact that these flowers are readily available and the extraction procedure is simple, with excellent performance, precise and accurate results would make a suitable substitute of presently available synthetic indicators.

In a nutshell, tannery industries, research laboratories, schools, and chemical companies that make use of indicators for the determination of acidity, alkalinity, humidity, extent of reactions, and so forth would find the preliminary results from this study valuable in producing efficient indicator from flowers as substitutes or possible replacement for standard indicators.

## Figures and Tables

**Table 1:** Percentage yield of the plant extract.

Plant	Extraction solvent	Mass of powder plant (g)	Mass of extract (dye) obtained (mg)	Percentage yield (%)
Rose	Water	20	220	1.10
	Methanol	20	325	1.63

Allamanda	Water	20	250	1.25
	Methanol	20	310	1.55

Hibiscus	Water	20	290	1.45
	Methanol	20	340	1.70

**Table 2:** Hibiscus extract (cold extract) (initial colour of extract, blood red).

Solvent	+	Extract	=	Colour change
ACID				
H_2_SO_4_	+	Extract	⇒	Pink
CH_3_COOH	+	Extract	⇒	Pink
BASE				
NaOH	+	Extract	⇒	Pale yellow
KOH	+	Extract	⇒	Pale yellow

pH = 5.24.

**Table 3:** Hibiscus extracts (methanol extract) (initial colour of extract: blood red).

Solvent	+	Extract	=	Colour change
ACID				
H_2_SO_4_	+	Extract	⇒	Pink
CH_3_COOH	+	Extract	⇒	Pink
BASE				
NaOH	+	Extract	⇒	Pale yellow
KOH	+	Extract	⇒	Pale yellow

pH = 6.52.

**Table 4:** Allamanda extracts (cold extract) (initial colour of extract, brown).

Solvent	+	Extract	=	Colour change
ACID				
H_2_SO_4_	+	Extract	⇒	Brown
CH_3_COOH	+	Extract	⇒	Brown
BASE				
NaOH	+	Extract	⇒	Yellow
KOH	+	Extract	⇒	Yellow

pH = 5.35.

**Table 5:** Allamanda extracts (methanol extract) (initial colour of extract, brown).

Solvent	+	Extract	=	Colour change
ACID				
H_2_SO_4_	+	Extract	⇒	Golden brown
CH_3_COOH	+	Extract	⇒	Golden brown
BASE				
NaOH	+	Extract	⇒	Yellow
KOH	+	Extract	⇒	Yellow

pH = 5.45.

**Table 6:** Rose extract (cold extract) (initial color of extract, red).

Solvent	+	Extract	=	Colour change
ACID				
H_2_SO_4_	+	Extract	⇒	Pink
CH_3_COOH	+	Extract	⇒	Pink
BASE				
NaOH	+	Extract	⇒	Yellow
KOH	+	Extract	⇒	Yellow

pH = 5.50.

**Table 7:** Rose extract (methanol extract) (initial color of extract, red).

Solvent	+	Extract	=	Colour change
ACID				
H_2_SO_4_	+	Extract	⇒	Pink
CH_3_COOH	+	Extract	⇒	Pink
BASE				
NaOH	+	Extract	⇒	Pale yellow
KOH	+	Extract	⇒	Pale yellow

pH = 5.60.

**Table 8:** Acid-base titration with extract as indicator.

Indicator	Strong acid (H_2_SO_4_)	Weak acid (CH_3_COOH)
	Strong base (NaOH)	Strong base (NaOH)
	cm^3^	cm^3^
Phenolphthalein	12.04 ± 0.15	41.80 ± 0.30
Methyl red	12.04 ± 0.05	44.20 ± 0.15
Bromophenol blue	12.06 ± 0.10	60.50 ± 0.05
Hibiscus (cold extract)	11.70 ± 0.06	25.00 ± 0.15
Hibiscus (methanol extract)	11.50 ± 0.25	25.50 ± 0.60
Allamanda (cold extract)	12.60 ± 0.20	42.60 ± 0.35
Allamanda (methanol extract)	12.00 ± 0.15	36.00 ± 0.50
Rose (cold extract)	13.00 ± 0.60	20.50 ± 0.45
Rose (methanol extract)	13.01 ± 0.40	22.60 ± 0.25

Values are mean ± SD of 3 readings.
